# Photodynamic Therapy Using HMME for Port-Wine Stains: Clinical Effectiveness and Sonographic Appearance

**DOI:** 10.1155/2020/6030581

**Published:** 2020-07-30

**Authors:** Ahmad Taha Khalaf, Yonghong Sun, Fang Wang, Minmin Sheng, Yan Li, Xiaoming Liu

**Affiliations:** ^1^College of Medicine (College of Nursing), Chengdu University, Chengdu 610106, China; ^2^Department of Nuclear medicine, The Third Affiliated Hospital of Soochow University, Changzhou, Jiangsu 213003, China; ^3^Department of Dermatology, The Third Affiliated Hospital of Soochow University, Changzhou, Jiangsu 213003, China; ^4^Department of Dermatology, Southern University of Science and Technology Hospital, Shenzhen, China 518055

## Abstract

This study aims at exploring the clinical efficacy and sonographic changes of photodynamic therapy (PDT) using Hematoporphyrin Monomethyl Ether (HMME) for the treatment of port-wine stains (PWS). Forty-five patients with PWS were recruited between March 2017 and June 2018 from the Department of Dermatology of The Third Affiliated Hospital of Soochow University. Five cases were of the pink type, thirty-nine cases were of the purple-red type, and one case was of the thickened type. All patients received three treatment sessions of PDT. After covering normal skin outside the treated area, patients received an intravenous injection of 5 mg/kg HMME within 20 minutes. The affected areas were exposed to a 532 nm LED light and were kept vertically at a distance of 10 cm. The irradiation energy density was set between 80 and 110 J/cm^2^ in 15-minute sessions. Intermittent power density adjustment was performed at a rate of 5 mW/cm^2^, and the treatment was withheld when the endpoint reaction appeared. Three follow-ups were performed before and after treatment, respectively, and the efficacy, thickness, and density of skin before and after treatment were evaluated with high-frequency ultrasound. The overall efficacy rate was 97.78% in forty-five cases after treatment for three sessions. Efficacy was related to age (*P* = 0.029) and lesion severity (*P* < 0.001). There were significant differences in the efficacy between the groups of <18 years old, 18-29 years old, and >29 years old (*P* = 0.029). A marked decrease in the numbers of distorted enlarged blood vessels per unit of the lesion was observed under high-frequency ultrasound. There were significant differences in skin thickness and skin density before and after treatment (*F* = 14.528, 5.428, *P* < 0.001). The swelling was reported to varying degrees in the treated areas in 23 patients with cheek lesion and in 6 frontal lesions. Hyperpigmentation after inflammation was observed in four patients that faded spontaneously after two months. In conclusion, photodynamic therapy for the treatment of PWS using HMME is effective and safe with few adverse reactions. Moreover, monitoring the changes in skin thickness and density of lesion tissue using high-frequency ultrasound can objectively evaluate the clinical efficacy of HMME photodynamic therapy and provide the basis for the formulation of individualized photodynamic therapy.

## 1. Introduction

A port-wine stain (PWS) is a congenital vascular malformation involving superficial dermis. The incidence is 0.3%-0.5%, which is usually on the face and neck [1]. Key biological pathways that lead to PWS are still not entirely clear. However, the blood vessels in PWS are immature capillary vessels with dual arterial-venous property; this ultimately evolves into venule-like vasculatures. Studies have shown a decrease in nerve density around the area of the dilated Vvessels, which may be responsible for an insufficient regulation of vascular tone. Vascular endothelial growth factor (VEGF) that regulates vascular proliferation and causes vasodilation with its receptors was abnormally overexpressed in the lesion also [2, 3]. Some researchers have postulated that PWS endothelial cells (ECs) are differentiation-impaired bone marrow–derived endothelial progenitor cells (EPCs). Thus, they are subject to a gradual expansion due to the disruption of normal EC-EC interactions through the coexistence of EphB1/EfnB2 [4].

A recent study using immunohistochemistry, immunoblot, and transmission electron microscopy (TEM) has shown that the extracellular vesicles released by lesional endothelial cells may act as possible intercellular signaling mediators in the pathogenesis of PWS. The lesion exhibited an upregulation of multiple proteins involving membrane trafficking and exocytosis in conjunction with enhanced blood vessel secretion of EVs [5]. Another study of the main pathological features and ultrastructure of cell types in hypertrophic and nodular PWS, through the TEM, has shown that the main features were the presence of hyperactive ECs, pericytes, and fibroblasts. This has a major role in the proliferation and replication of the stroma, which may lead to the hypertrophic and nodular types of PWS [6].

PWS generally does not fade naturally. With the increase of age, the color will deepen and become purple. About 70% of patients will gradually progress to show thickening with some nodules that may lead to localized destruction [7]. Therefore, if PWS patients receive early intervention, they can improve their quality of life and avoid the occurrence of complications and disfigurements. PWS can be divided into pink, purplish-red, and nodular thickening types. At present, the pulsed dye laser (PDL) is the standard clinical treatment method for almost all age groups. However, only a small number of patients can achieve complete clearance while about 20%-30% of patients show resistance to treatment with PDL [8, 9]. Photodynamic therapy has been widely used in China to treat PWS since the early 1990s. The treatment can selectively destroy vascular endothelial cells, coagulate blood vessels, lead to fibrosis, and ultimately enhance the removal of the deformed expanded capillary network of the skin. Thus, these diseases can be effectively treated while maintaining the natural appearance of the skin. Hematoporphyrin Monomethyl Ether (HMME) has been used as a new type of porphyrin monomer optical photosensitizer, widely used in the treatment of microvascular malformations in recent years due to its high photodynamic efficiency, strong photoactivity, and rapid clearance rate. Several experimental studies have shown that the use of HMME is safe and effective in photodynamic therapy. It has a stable and low-toxic formula and is relatively inexpensive [10, 11].

The average depth of PWS is 0.46 + 0.17 mm, and as the energy density of the laser decreases with increasing skin thickness, so in this study, and after the intravenous injection with HMME, as a photosensitizer, we used a laser instrument with a penetration depth of about 400 nm and with a 532 nm LED instrument with a continuous power output and a wavelength close to the length of the HMME. At the peak of HMME absorption, this wavelength can effectively activate the photosensitizer without damaging the surrounding tissue [9–11]. Before and after HMME dynamic photodynamic therapy, the thickness and density of the skin and blood vessels of the PWS were measured under high-frequency ultrasound, the clinical efficacy of HMME was assessed, and adverse reactions were monitored.

## 2. Patients and Methods

### 2.1. Participants

From March 2017 to June 2018, patients with clinically confirmed PWS were recruited at the Dermatology Clinic of The Third Affiliated Hospital of Soochow University.

### 2.2. Inclusion Criteria

Patients with PWS who met the diagnostic criteria [11] and were aged 6 to 37 years. A voluntary signature of informed consent was obtained from all participants. For patients under the age of 18, the guardian's signature was obtained to sign informed consent at the same time.

### 2.3. Exclusion Criteria

(1) Porphyria patients or those with a known allergic history of porphyrins and related substances. (2) Patients with heart, liver, kidney, chronic, and other systemic diseases or those who use anticoagulants. (3) Pregnancy and lactation. (4) Those who have interference factors affect the absorption and distribution of the drug, metabolism, and secretion. (5) Those who do not follow the doctor's instructions and fail to complete the course of treatment due to various factors during treatment. (5) Those who fail to follow-up on schedule. This study was approved by the Medical Ethics Committee of Changzhou First People's Hospital (Approval No. (2018) Section 037 (Rapid Trial)).

### 2.4. Clinical Data

The study included 45 patients with PWS, including 9 males and 36 females. The participants ranged in age from 6 to 37 years old. Six were under 18 years old, 30 were between 18 and 29 years old, and nine were over 29 years old. The lesions were distributed as follows: 4 cases in the cheek, 8 cases in the frontotemporal area, 8 cases in the lower jaw and chin, 2 cases in the middle of the face, and 3 cases in other parts of the body (including the trunk and limbs). Skin types by reference to the Fitzpatrick classification were III and IV, and according to the color and thickening of the lesion, PWS was divided into four types: pink type (5 cases), purple type (39 cases), thickened type (1 case), and nodular type (1 case) (see Table 1). Among 45 patients, 24 (53.33%) received more than one type of treatment, 23 (51.11%) were laser treated, and 1 (2.22%) was treated with PDT. There were 6 cases with a previous lesional scar (13.33%) and two cases with glaucoma (4.44%).

### 2.5. Therapeutic Drugs and Methods

(1) HMME was produced by Shanghai Fudan Zhangjiang Biopharmaceutical Co., Ltd., China (The pharmaceutical batch number was H20120076); (2) Protection of normal skin: after determining the lesion site, we covered the normal skin with a double-layer red and black cloth around the skin lesion to avoid light; (3) Skin test: to make sure there was no sensitivity, the diluted solution of HMME was injected intradermally in one forearm, with normal saline used for control. Patients with the negative results only used HMME; (4) Intravenous administration: after the establishment of the venous access, dexamethasone (2-3 mg in children, 5-10 mg in adults) was given intravenously to prevent drug allergic reactions during the administration and to relieve the treatment reaction. The intravenous infusion pump was set to a rate of 150 ml/h with an injection dose of HMME at 5 mg/kg in a dark room. The injection of HMME was diluted with 50 ml of normal saline and was given over 20 minutes; (5) Light source setting: the light source plate was set parallel to the processing surface, and the distance between each point of the treatment surface and each point of the light source plate was fixed to be 10 cm. Intravenous infusion with HMME was first done for 10 minutes and then to the light treatment with PDT. The LED PDT therapeutic machine (LED-IE, Wuhan Yage Optoelectronics Technology Co., Ltd.) was set to a wavelength of 532 nm between 80 and 110 J/cm^2^. The energy density and skin temperature were assessed during therapy. After 15 minutes of therapy, the light was paused, and the treatment response was observed. The lighting parameters were adjusted according to the local reaction, and light intensity was adjusted by 5 mW/cm^2^ until a suitable treatment endpoint reaction, such as dark purple, gray, or fading.

### 2.6. End of Treatment Observation

During the second half of the light exposure, attention was timely paused to observe the local reaction of the treatment surface, such as the treatment endpoint reaction like purpura in the existing treatment area, and protect these areas with an adhesive tape to avoid excessive lighting. In the light range, special parts, such as the nose, nasolabial folds, and lip angles were covered with tape after 15 minutes of light exposure to avoid thick sputum. If there was severe pain during the light exposure, the patient could request a pause.

### 2.7. Prevention and Management of Treatment-Related Reactions

(1) An immediate cold spraying or cold compress was used after each treatment for 20 minutes time; (2) Attention was pay to eye protection during treatment, especially children. In addition, the patient was advised to avoid strong direct light source within two weeks after treatment; (3) When the treatment area was large or involves areas that were prone to swelling, glucocorticoid intervention was used after treatment; (4) The patient was advised not to touch, scratch, or expose the treated area to water within a week of treatment. If the treated area was relatively dry, moisturizing cream was used; (5) Strenuous exercises were prohibited until the treatment effect is stable; (6) Patients were informed about the possibility of treatment-related reactions so that the patients have some psychological preparation.

### 2.8. High-Frequency Ultrasonic Measurement Evaluation

The skin of the part to be measured was fully exposed, cleaned, and disinfected. The frequency in the high-frequency ultrasonic measuring instrument (GE-LOGIQ-E9; GE American; Probe Model ML6-15) was set to 15 MHz and the penetration depth to 10 mm. The thickness and density of the skin were measured in the lesions as well as on the normal skin on the opposite sides of the skin lesions. Semiquantitative radiographic assessment and grading were performed on the order of grade 0-3. The same part was measured three times, and the average value was taken. All data and ultrasound images were stored on the computer.

### 2.9. Efficacy Criteria

Image collection of skin lesions in patients was done with the same SLR digital camera, angle, and light source. Three senior dermatologists in our hospital independently compared the changes in skin lesions before and after treatment. Cure: the color of the lesion subsided by more than 90%, close to or the same as normal skin; Markedly effective: the color of the lesion subsided by about 60-90%, the thickened part flattened with normal skin; Effective: the color of the lesion subsided by about 30-60%, the thickening part is obviously thinner and close to normal skin; Ineffective: the color of the lesion did not improve and the thickened part had no obvious changes.

### 2.10. Statistical Analysis

Statistical analysis was performed using SPSS 20.0 software. The count data were expressed by rate. The subjective evaluation part of the grade data was calculated according to different scoring standards. The median and interquartile range was used, and data were analyzed by the nonparametric test. The Mann-Whitney *U* test and Kruskal-Wallis test were applied. The measurement data were done using a one-way analysis of variance, and the comparisons between groups were performed by the Student–Newman–Keuls (SNK) test. *P* value < 0.05 was considered to indicate statistical significance.

## 3. Results and Discussion

### 3.1. Observation of the Efficacy of HMME-PDT in the Treatment of PWS

**Figure 1:**
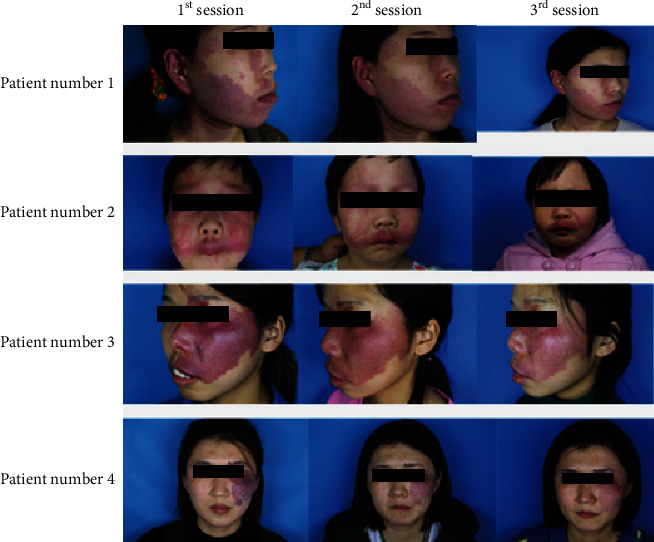
Comparison of the efficacy of HMME-PDT in the treatment of PWS, after three treatments. Case 1: 35-year-old female patient with pink lesions under the left orbit, back of the nose, cheeks, and upper lip. After three HMME-PDT treatments, the patient's cheek skin gradually changed from pink to light red, and the suborbital, dorsal nasal, and upper lip skin lesions basically subsided. Case 2: a 24-year-old female patient with bright red patches on the right iliac temporal, cheek, nose, and upper lip, and slight swelling of the right lip corner. After three HMME-PDT treatments, the bright red patches on the lesions subsided by about 65%, from bright red to light red, mild pigmentation of the crotch skin. Case 3: 26-year-old female patient with purple patches on the left parotid gland and mandible with purple-red lesions. She had had hypertrophic scars in the mandible after laser treatment. After three HMME-PDT treatments, the lesions subsided about 80%. Case 4: a 28-year-old female patient with dark purple patches on the left orbital condyle and cheeks, with a slightly thickened skin surface. After three HMME-PDT treatments, the color of the skin lesions gradually changed from dark purple to pale red, the skin at the thickened area was basically flat.

**Figure 2:**
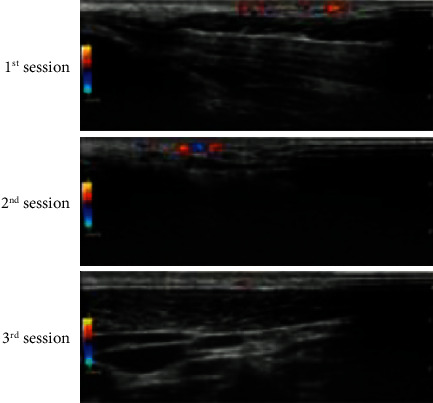
Changes in skin thickness and density before and after treatment with PWS in the high-frequency ultrasound skin mode. Before treatment, compared with the normal skin on the opposite side, the dilated deformed capillaries in PWS patients increased significantly, from the superficial layer of the dermis to the deep layer. After three HMME-PDT treatments, the deformed vessels in the superficial dermis of the skin lesions were significantly reduced, and a small number of star-shaped blood flow signals were seen.

Compared with contralateral normal skin, patients with pink, purple-red, and thickened PWS had significantly more dilated malformed capillaries, expanding from the superficial layer to the deeper layer of the dermis (see Figure 1). After 3 times of HMME-PDT treatment, compared with before treatment, the deformed blood vessels in the unit area of the patient were significantly reduced, new blood vessels appeared, and a small number of star-shaped blood flow signals were seen, indicating that a considerable number of deformed blood vessels in the superficial dermis were damaged (see Figure 2). Of the 45 PWS patients who completed the treatment and were followed up, 10 cases were cured, 21 cases were markedly effective, 13 cases were effective, and one case was ineffective. The total effective rate was 97.78%. The Mann-Whitney *U* test showed no significant difference in efficacy between different genders (*P* = 0.244). The Kruskal-Wallis test showed that there was a significant difference in efficacy between different age groups (*P* = 0.029). Compared with the 18-year-old group and the 18-29-year-old group and the >29-year-old group, the 18-year-old group had the best effect (*H* = −31.50, -121.08, *P* < 0.05); there was no significant difference in efficacy between different lesion distributions (*P* = 0.363). Because there is only one case of thickening type in the degree of lesions, it is not involved in statistical analysis. After the Mann-Whitney *U* test, there is a significant difference in efficacy between pink and purple-red lesions (*P* < 0.001). The curative effect was related to age (*P* = 0.029), and the effect was best in the <18-year-old group; it was also related to the degree of disease (*P* < 0.001), and the pink type had the best curative effect (see Table 2).

### 3.2. High-Frequency Ultrasound Measurement of PWS Skin Lesions after HMME-PDT Treatment

Under high-frequency ultrasound skin mode, the thickness and density of normal skin on the contralateral side were measured before and after the treatment of PWS patients. Because there was only one case in the ineffective group, it did not participate in the statistical analysis. There were significant differences in the skin thickness before and after the treatment of three types of skin lesions with different curative effects after one-way analysis of variance (*F* = 14.52853, *P* < 0.001). Through the SNK test, there was a significant difference between the cured group and the marked effective group; also, between the marked effective group and the effective group. The difference in skin density between the three curative effects before and after treatment was significantly different (*F* = 5.42843, *P* = 0.008). There was a significant difference in the thickness of the skin between the cured group and the effective group greater than the effective group (*q* = 7.22, 5.82, *P* < 0.05). The difference between the curative group and the effective group was statistically significant (*q* = 4.63, *P* < 0.05) (see Table 3).

### 3.3. HMME-PDT Treatment Adverse Event Monitoring Events

45 patients with PWS showed different degrees of pain at about 5 minutes of light. Different degrees of pain appeared at about min. Pauses of light and cold wind were used if needed. Although there was still some pain, in general, it did not affect subsequent treatment. Treatment of adverse reactions: 23 patients with the palate, cheek, and 6 frontotemporal areas had different degrees of redness and swelling in the light area after treatment. They were given immediate cold spray or cold compress and corresponding symptomatic treatment. Generally, the redness and swelling gradually subsided in about one week. No adverse reactions were observed in nonlight areas. Light brown crusts appeared in 35 patients with PWS, and crusts fell off on their own in about three weeks. Four patients with PWS developed pigmentation after inflammation in the light site. However, the pigment gradually faded. Forty-five PWS patients did not develop a hypertrophic scar. No adverse reactions were observed in nonlight areas.

The type of photosensitizer plays a decisive role in the efficacy of photodynamic therapy. Diffusion from blood vessels to tissues leads to the production of phototoxic substances and tissue damage; ultimately, photosensitive bleaching is an important physical and chemical process for photodynamic therapy [11]. So far, the most commonly used and under study photosensitizers are HMME, hematoporphyrin derivative (HpD), and PsD-007. HMME is a new type of photosensitizer. Compared to the first-generation photosensitizers (such as HpD), its chemical composition is stable with a relatively low porphyrin content and other advantages. At present, HMME is mainly developed as a photosensitizer for PDT treatment. Because of its characteristic of having a long life and strong fluorescence, its potential in photodynamic diagnosis is also attracting attention [12, 13].

The results of this study found that the total effective rate of HMME-PDT treatment of PWS was 97.78%. There were differences at different ages (*P* = 0.029). This may be related to the relative increase in the number and diameter of deformed blood vessels in patients with increasing age. The therapeutic effect of patients with different degrees of disease also has differences (*P* < 0.001). The skin lesions of pink type are superficial, the diameter of the deformed blood vessels is relatively small, and the response to treatment is relatively good, which is consistent with the results of other studies [14]. There is no significant difference in the efficacy of different lesion distributions. This is inconsistent with previous research results and may be related to the small sample size in this study. This study found that the main adverse reactions of HMME-PDT were pain, swelling, crusting, and pigmentation, which subsided to varying degrees after timely treatment. However, whether there is a correlation between treatment adverse reactions and treatment effects still needs further research. In addition, it should be noted that during the treatment process, intermittent monitoring of power density, skin temperature, and timely pausing is required to observe the local reaction of the treatment to avoid skin necrosis and other reactions [15–17].

Ultrasound imaging has been used in the diagnosis of skin diseases since the late 1970s. With the continuous development of high-frequency ultrasound instruments, the resolution of high-frequency ultrasound continues to increase. It can be used not only to measure skin thickness, but also to detect skin tissue density, size, blood flow changes, and skin appendages [9]. It is widely used in the diagnosis and evaluation of skin tumors, psoriasis, scleroderma, and acne scars [16–21]. The most commonly used ultrasound frequency for researching skin diseases is about 15-20 MHz, with a penetration depth of 10 mm and a resolution of more than 0.1 mm. It can clearly show the tissue structure of the skin epidermis and dermis. In theory, high-frequency ultrasound technology can meet the PWS Measurement and assessment of skin lesions in patients. In this study, we used 15 MHz high-frequency ultrasound to measure the thickness and density changes of the normal skin and lesions on the contralateral side of PWS patients before and after treatment and initially discussed the feasibility of using high-frequency ultrasound to judge the effect of PWS treatment [21–24]. We found that after photodynamic therapy with hempofen, the skin thickness of PWS patients became thinner and denser. The skin thickness changes in the healing group and the markedly effective group were the most obvious, while the skin density changes in the healing group were more significant than those in the effective group. This shows that the efficacy of HMME-PDT treatment is closely related to the thickness and density of skin lesions. High-frequency ultrasound, as a noninvasive and highly reproducible examination method, measures skin thickness and density changes and is morphologically comparable to pathological features to a certain extent. It can be used as a PWS patient to evaluate clinical outcomes after HMME-PDT treatment. In subsequent studies, we plan to include more research data to compare and analyze skin lesion thickness and density changes before and after treatment of PWS patients with different ages, different lesion distributions, and different degrees of lesions, in order to build a more comprehensive objective evaluation plan for the efficacy of PWS.

## 4. Conclusion

Photodynamic therapy is currently one of the most promising methods for the treatment of PWS, but it is also a technique with operations that are more complicated and many influencing factors. The treatment process needs to be further standardized in order to ensure its efficacy and safety.

## Figures and Tables

**Table 1:** Patient demographics.

Clinical characteristics	Number of cases (%)	Lesion area cm^2^ (^`^*x* ± *s*)
Sex		
Male	9 (20.00)	18.14
Female	36 (80.00)	20.98
Age		
<18 years old	6 (13.33)	3.65
18 to 29 years old	30 (66.67)	18.12
>29 years old	9 (20.00)	22.36
Lesion distribution		
Cheek	24 (53.33)	29.21
Frontotemporal	8 (17.78)	14.14
Lower jaw and palate	8 (17.78)	17.92
Middle face	2 (4.44)	10.01
Other parts	3 (6.67)	14.26
Degree of disease		
Pink type	5 (11.11)	13.13
Purple-red	39 (86.67)	22.12
Thickened	1 (2.22)	34.15

**Table 2:** Observation on the efficacy of HMME-PDT in the treatment of PWS.

	Number of cases	Efficacy assessment				Statistics	
		Cured	Marked effective	Effective	Ineffective		
Gender						*U* = 120.50	*P* = 0.244
Male	9	1 (11.11)	4 (44.44)	3 (33.33)	1 (11.11)		
Female	36	9 (25.00)	17 (47.22)	10 (27.78)	0 (0.00)		
Age						Hc = 7.09	*P* = 0.029
<18	6	4 (66.67)	1 (16.67)	1 (16.67)	0 (0.00)		
18 to 29	30	4 (13.33)	16 (53.33)	10 (33.33)	0 (0.00)		
>29	9	2 (22.22)	4 (44.44)	2 (22.22)	1 (11.11)		
Lesion distribution						Hc = 4.33	*P* = 0.363
Cheek	24	5 (20.83)	14 (58.33)	5 (20.83)	0 (0.00)		
Frontal	8	2 (25.00)	3 (37.50)	3 (37.50)	0 (0.00)		
Mandibular and Ankle	8	3 (37.50)	2 (25.00)	3 (37.50)	0 (0.00)		
Middle	2	0 (0.00)	0 (0.00)	2 (100.00)	0 (0.00)		
Other parts	3	0 (0.00)	2 (66.67)	0 (0.00)	1 (33.33)		
Degree of lesion						*U* = 12.50	*P* < 0.001
Pink type	5	5 (100.00)	0 (0.00)	0 (0.00)	0 (0.00)		
Purple red	39	5 (12.82)	21 (53.85)	13 (33.33)	0 (0.00)		
Thickened	1	0 (0.00)	0 (0.00)	0 (0.00)	1 (100.00)		

Notes: the efficacy of HMME-PDT in the treatment of PWS was observed by the Mann-Whitney *U* test. There was no significant difference between the sexes (*P* = 0.244). The Kruskal-Wallis test showed that there were significant differences in the efficacy among different age groups (*P* = 0.029). The group under 18 years old had the best efficacy, and there was no significant difference in the efficacy between different lesion distribution (*P* = 0.363). Because there was only one case of the hypertrophic type, it was not involved in the statistical analysis. The Mann-Whitney *U* test showed that there were significant differences in the curative effect among different degrees of lesion (*P* < 0.001). The pink type showed the best curative effect, followed by the purple-red type.

**Table 3:** Changes in skin thickness and density of patients with PWS before and after of HMME-PDT (*x* ± *s*).

		Skin thickness (mm)			Skin density (g/cm^3^)				
Efficacy	Number of cases	Normal skin	Before treatment	After treatment	Difference	Normal skin	Before treatment	After treatment	Difference
Cured	10	1.33	1.98	1.23	0.74	22.04	13.00	21.69	-8.72
Sign. Eff.	21	1.12	1.67	1.09	0.60	28.49	14.88	25.47	-6.95
Effective	13	1.45	1.69	1.42	0.33	29.31	12.12	25.13	-4.85
Ineffective	1	1.47	1.77	1.61	0.16	24.52	15.04	17.23	-2.19
*F* value					14.53				5.43
*P* value					<0.001				0.008

## Data Availability

The data used to support the findings of this study are available from the corresponding author upon request.
